# Synthetic Strategies for Dinucleotides Synthesis

**DOI:** 10.3390/molecules24234334

**Published:** 2019-11-27

**Authors:** Lucie Appy, Crystalle Chardet, Suzanne Peyrottes, Béatrice Roy

**Affiliations:** Nucleosides & Phosphorylated Effectors, Institut des Biomolécules Max Mousseron (IBMM), UMR 5247 CNRS, Université de Montpellier, ENSCM, Campus Triolet, cc 1705, Place Eugène Bataillon, CEDEX 5, 34095 Montpellier, France; lucie.appy@gmail.com (L.A.); crystalle.chardet@gmail.com (C.C.); suzanne.peyrottes@umontpellier.fr (S.P.)

**Keywords:** phosphorylation, dinucleotides, organophosphorus chemistry, mechanochemistry, dry eye syndrome, diquafosol, denufosol

## Abstract

Dinucleoside 5′,5′-polyphosphates (DNPs) are endogenous substances that play important intra- and extracellular roles in various biological processes, such as cell proliferation, regulation of enzymes, neurotransmission, platelet disaggregation and modulation of vascular tone. Various methodologies have been developed over the past fifty years to access these compounds, involving enzymatic processes or chemical procedures based either on P(III) or P(V) chemistry. Both solution-phase and solid-support strategies have been developed and are reported here. Recently, green chemistry approaches have emerged, offering attracting alternatives. This review outlines the main synthetic pathways for the preparation of dinucleoside 5′,5′-polyphosphates, focusing on pharmacologically relevant compounds, and highlighting recent advances.

## 1. Introduction

**Figure 1:**
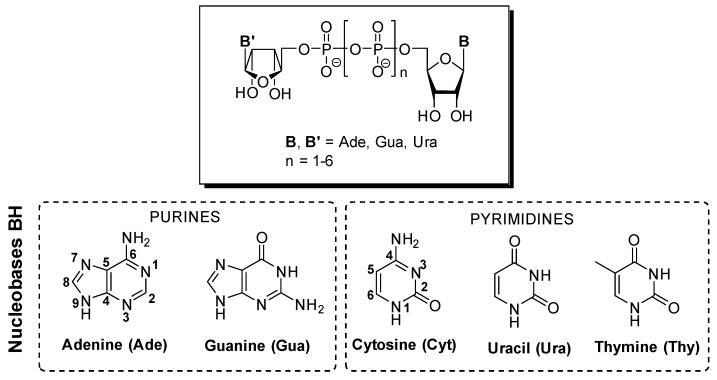
General structure of natural dinucleoside 5′,5-polyphosphates and canonical nucleobases.

Dinucleoside 5′,5′-polyphosphates (DNPs), commonly abbreviated as Np*_n_*Ns, are essential to human biological systems [1,2]. They contain two ribonucleosides, which are linked at the 5′-position of their sugar moiety through *n* phosphate groups (Figure 1).

Historically, dinucleoside 5′,5′-polyphosphates (i.e., Ap_2_A and Up_2_U) were first isolated by chemists [3]. In 1976, E. Rapaport and P.C. Zamecnik brought to light the presence of *P*^1^,*P*^4^-diadenosine-5′-tetraphosphate (Ap_4_A) in mammals [4]. The authors assumed that this dinucleotide could have a role in cellular communication as a “signal nucleotide”. Later, Up_4_A was the first endogenous dinucleotide, possessing both purine and pyrimidine moieties, to be identified in living organisms [5,6]. Currently, 17 symmetrical or mixed Np*_n_*Ns have been isolated from human tissues and characterized [2]. DNPs are released into the extracellular space from different types of cells, such as platelets and endothelial cells, where they stimulate several cell-surface purinergic receptors. They have a strong physiological and pathophysiological impact on the cardiovascular system [2,7], and may also interact with enzymes, acting as inhibitors (e.g., kinases) or substrates [8,9,10,11]. Three classes of enzymes are known to catalyze the degradation of dinucleotides [10,11,12]: symmetrically cleaving dinucleoside polyphosphate hydrolases, asymmetrically cleaving dinucleoside polyphosphate hydrolases, and dinucleoside polyphosphate phosphorylases. Over the years, DNPs have gained increased attention and their therapeutic potential has been revealed. Likewise, structural analogues have been developed and some of them are drug candidates.

Herein, we first describe purinergic signalling, i.e., the role of nucleotides as extracellular signal molecules, with a focus on the P2Y receptor subtypes that interact with dinucleotides. Then, we present the synthetic strategies to obtain dinucleotides. Pyrophosphate bond formation has been addressed in some recent reviews and book chapters [1,13,14,15]. The current review focuses on methods reported for the synthesis of dinucleoside 5′,5′-polyphosphates, especially those of biological and pharmacological interest. We also highlight recent advances in the field, such as green chemistry approaches. It should be noted that methods for preparing cyclic dinucleotides [16] are beyond the scope of this review. Furthermore, the synthesis of nicotinamide adenine dinucleotides [17] and mRNA cap analogues such m^7^Gp*_n_*N (see reviews [18,19,20]) are not presented.

## 2. Interaction of DNPs with Purine and Pyrimidine Receptors

**Figure 2:**
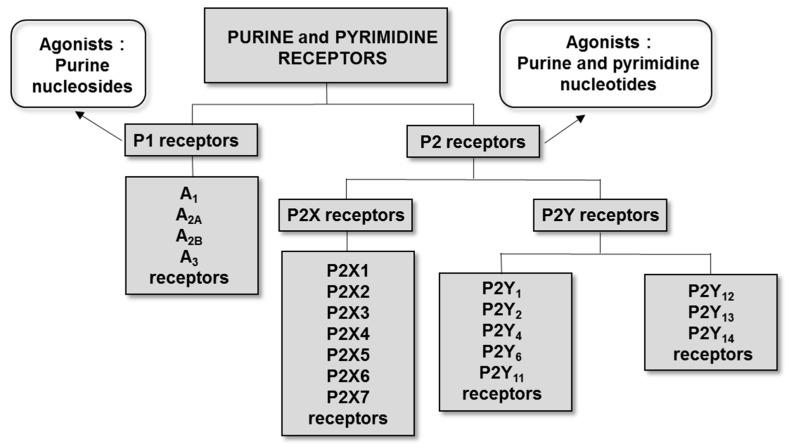
The classification of purine and pyrimidine receptors and their main physiological agonists.

Purinergic receptors are extracellular receptors, currently consisting of four subtypes of P1 (or adenosine) receptors, seven subtypes of P2X ion channel receptors, and eight subtypes of P2Y receptors (Figure 2). Specifically, P2Y receptors (P2YRs) are G-protein-coupled receptors (GPCRs) activated by extracellular nucleotides, which are divided into two groups on the basis of sequence homology and the type of G protein they are primarily coupled to [21,22,23].

Purinergic receptors are expressed in nearly all cell types, and have been found to play crucial roles in many biological processes, e.g., neurotransmission, neuroprotection in hypoxia and ischemia, regulation of cardiovascular function, platelet aggregation, smooth muscle contraction, secretion of hormones, modulation of immune response, control of cell proliferation, differentiation, and apoptosis. In this regard, different aspects of purinergic signaling such as the pathophysiology and the therapeutic potential have been extensively reviewed [21,22,23,24,25,26,27,28]. Several purinergic compounds are already on the market to treat thrombosis and stroke, and one dinucleotide is used to treat dry eye disease (see Section 2.1).

**Figure 3:**
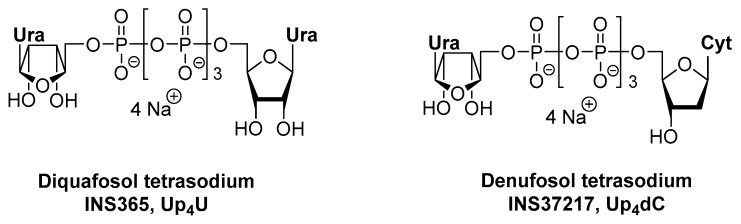
Dinucleotides with clinical developments.

DNPs are strong endogenous agonists of the purinergic system [2]. In particular, they interact with the P2YRs, a family of high therapeutic relevance [2,23,29,30]. The P2Y_1_, P2Y_11_, P2Y_12_, and P2Y_13_ receptors are activated by adenosine 5′-di- or triphosphates (ADP and ATP), while uracil nucleotides (UDP and UTP) are the endogenous agonists for the P2Y_4_, P2Y_6_, and P2Y_14_ receptors. On the other hand, the P2Y_2_ receptor responds to both ATP and UTP. Natural dinucleoside tetraphosphates Ap_4_A and Up_4_U have agonist potencies comparable to those of ATP and UTP at P2Y_2_ receptors [31,32,33,34]. It should be noted that dinucleotides are more resistant to hydrolysis than their parent mononucleotides, but are generally less potent, and lack selectivity in many cases [9,23]. Ligand development for the class of uracil nucleotide-activated P2Y receptors has been extensively reviewed by Rahefi and Müller in 2018 [23]. They have compiled existing data on, inter alia, dinucleotides such as Ap*_n_*A (*n* = 2–6), Up*_n_*U (*n* = 3–4) and structural analogues, as agonists for the uracil-activated P2YRs. Given the significant clinical potential of the P2YRs, substantial research efforts directed towards developing P2YR ligands for use as pharmacological tools and drugs have led to the discovery and development of a significant number of agonists but, so far, only a moderate number of antagonists. Two relevant dinucleotides, namely Up_4_U and Up_4_dC, have undergone clinical development to cure several diseases involving the P2Y_2_ receptor (Figure 3). 

### 2.1. Up_4_U for the Treatment of Dry Eye Disease (DED)

Diquafosol tetrasodium (*P^1^*, *P^4^*-diuridine 5′,5′-tetraphosphate, Up_4_U, NS365, *Diquas*^®^) is the first agonist of the P2Y_2_ receptor subtype that has been approved in Japan as 3% ophthalmic solution (Santen Pharmaceutical Co, Ltd., Osaka, Japan) for the management of dry eye disease (see reviews by Lau et al. [35], and Keating [36]). This pathology commonly causes symptoms including dryness, irritation, itching, and light sensitivity. It is associated with tear film instability, increased tear film osmolarity, and ocular surface inflammation. It impacts the daily life of patients and has a prevalence that varies from 5% to 35%. DED results from either decreased aqueous tear production (aqueous tear-deficient dry eye) or increased tear evaporation (evaporative dry eye), or both. Several studies demonstrated the presence of P2X and P2Y receptors in ocular tissues (retina, ciliary body, and lens), and indicated that P2Y_2_ receptors may be the main subtype of purinergic receptor located at the ocular surface [33]. Clinical data show that diquafosol tetrasodium improves ocular surface staining and may improve tear film volume and stability.

### 2.2. Up_4_dC for the Treatment of Cystic Fibrosis

Denufosol tetrasodium (*P^1^*-uridine-*P^4^*-2′-deoxycytidine 5′,5′-tetraphosphate, Up_4_dC, dCp_4_U, INS37217) is a mixed dinucleoside tetraphosphate. Along with Ap_4_A and Up_4_U, dCp_4_U acts as a P2Y_2_ agonist [29,37]. It has been developed by Inspire Pharmaceuticals (now part of Merck) as an inhaled drug for the treatment of cystic fibrosis [38]. This recessive genetic disease is characterized by pulmonary and reproductive tract dysfunctions, involving abnormal ion transport and defective mucociliary clearance. Denufosol acts on P2Y receptors expressed on the surface of airway epithelia to stimulate chloride secretion independent of the chloride channel, which is dysfunctional in cystic fibrosis. It was shown to significantly enhance tracheal mucus transport in an animal model [39,40]. The first clinical trials established a good safety profile. It was evaluated in two phase 3 clinical trials, TIGER-1 and TIGER-2, as a therapy for cystic fibrosis patients [41,42]. In the first trial (TIGER-1), it was found to significantly improve lung function in cystic fibrosis patients with normal to mildly impaired lung function. Unfortunately, less than 3 weeks after publication of the TIGER-1 data, Inspire Pharmaceuticals announced that the 466-patients, 48-week placebo-controlled phase 3 TIGER-2 clinical trial, had failed to demonstrate any benefit [41,42].

## 3. Synthesis Based on P(V) Chemistry

To date, the most widely used methods to access dinucleotides involve P(V) chemistry. They are based on the activation of a 5′-nucleotide (i.e., nucleoside 5′-mono, 5′-di and 5′-triphosphate) and the reaction of the corresponding intermediate with a second 5′-nucleotide or inorganic pyrophosphate to form dinucleotides containing up to six bridging phosphate groups. These steps are usually performed in dry, aprotic solvents (mostly *N*,*N*-dimethylformamide, DMF), and, therefore, require the use of nucleotides, as well as inorganic phosphate or pyrophosphate, in their tri- or tetra-*n*-butylammonium forms due to solubility issues. Divalent cations, especially Mg^2+^ and Zn^2+^, are sometimes added as catalysts for anhydride bond formation. It is assumed that the metal ion could serve two roles, namely, the activation of the electrophilic P(V) center and templating the incoming phosphate nucleophile and the P(V) electrophilic center [13].

### 3.1. Synthesis via a Phosphoromorpholidate Intermediate

**Scheme 1:**
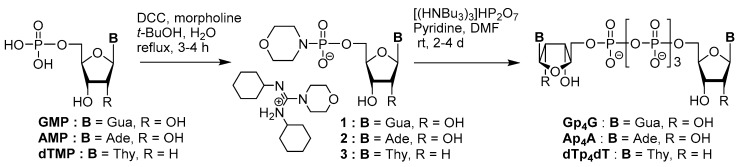
Synthesis via phosphoromorpholidate intermediates [44].

The first description of a chemical synthesis of dinucleotides was reported in the mid-60s by Moffatt and Khorana [43,44]. This strategy relies on the conversion of nucleoside 5′-monophosphates (NMPs) into their phosphoromorpholidate derivatives, followed by reaction with a phosphate salt, i.e., orthophosphate or pyrophosphate (Scheme 1). Briefly, the acidic form of the nucleotides were activated with N,N’-dicyclohexylcarbodiimide (DCC) in the presence of morpholine, leading to the nucleoside 5′-phosphoromorpholidates as 4-morpholine N,N’-dicyclohexylcarboxamide salts **1–3** (Scheme 1) [45]. In the second step, addition of pyrophosphate afforded the symmetrical dinucleoside tetraphosphates. Similarly, dinucleoside triphosphates were obtained when pyrophosphate was replaced by orthophosphate. 

Since then, this approach has been adapted by several research groups to access Np_2_N, Np_3_N, as well as some analogues. For example, Ap_2_A was obtained in 90% yield, through the condensation of the bis-(tri-*n*-butylammonium) salt of AMP and the adenosine-5′-phosphoromorpholidate-4-morpholine-N,N’-dicyclohexylcarboxamide salt in anhydrous pyridine [46]. Mixed dinucleoside triphosphates were obtained in 10–30% yields, by reacting nucleoside 5′-phosphoromorpholidates with nucleoside 5′-diphosphates (NDPs), possibly in the presence of tetrazole [31,47,48]. However, due to long reaction times and generally low yields, this method has lost its relevance.

### 3.2. Synthesis via a Phosphorimidazolide Intermediate

**Scheme 2:**
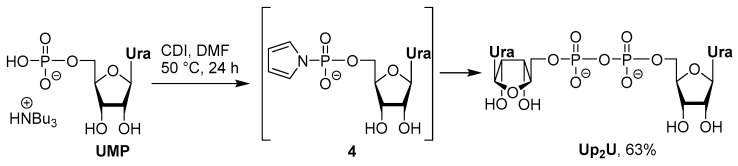
Activation of UMP by CDI to access Up_2_U [47,49].

Phosphorimidazolide derivatives exhibit high reactivity toward various nucleophiles. Therefore, they have been extensively used as intermediates for pyrophosphate bond formation. Typically, activation of a 5′-nucleotide (NMP, NDP or NTP) with *N*,*N*’-carbonyldiimidazole (CDI) in DMF, followed by the in situ condensation with a second 5′-nucleotide or pyrophosphate, affords dinucleotides containing two to four bridging phosphate groups in 10–60% yields [47,48,49,50]. Accordingly, the tri-*n*-butylammonium salt of UMP was activated with CDI to form a phosphorimidazolide intermediate **4**, which reacted with the remaining nucleotide to form Up_2_U (Scheme 2) [47,49]. Slight modifications of this protocol allowed access to mixed dinucleotides [50]. First, activation of the bis(tri-*n*-butylammonium) salt of CMP was performed using ≈ 2 equiv of CDI in DMF for 2 h at rt, followed by the addition of dry methanol to quench the remaining CDI. Treatment with the bis(tri-*n*-butylammonium) salt of GMP for 2 h at rt afforded Gp_2_C in 54% yield. Addition of GDP instead of GMP in the second step afforded Gp_3_C, albeit in only 3% yield. Similarly, activation of NDPs with CDI followed by reaction with a NMP allowed the isolation of dinucleoside triphosphates in low yields [48].

**Scheme 3:**
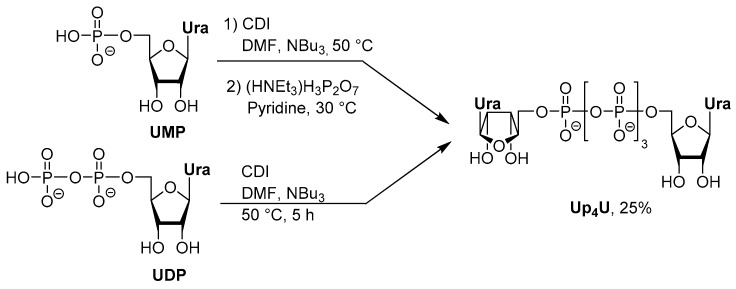
Dimerization of nucleotides to access symmetrical Up_4_U [49,51].

Alternatively, symmetrical dinucleoside tetraphosphate Up_4_U was obtained, either by activation of UMP with CDI followed by coupling with bis(tri-*n*-butylammmonium) pyrophosphate [47,49] or dimerization of UDP in the presence of CDI [51] (Scheme 3).

**Scheme 4:**
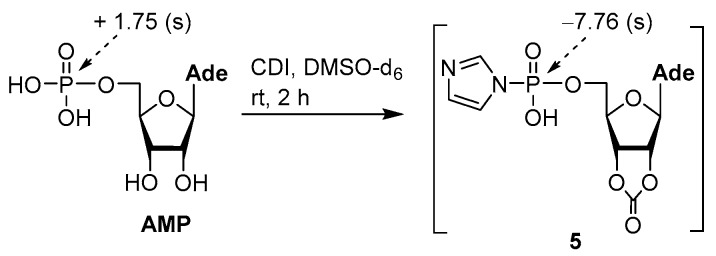
Reaction of AMP with 4 equiv CDI [52]. ^31^P chemical shifts are indicated in ppm together with their multiplicity.

Activation of ribonucleotides in the presence of an excess of CDI usually results in the carbonation of the ribose moiety (Scheme 4). In ^1^H-decoupled ^31^P NMR spectroscopy, adenosine 5′-phosphorimidazolide is characterized by a singlet at −7.56 ppm, whereas phosphorimidazolide **5** exhibits a singlet at −7.76 ppm [52]. However, in some of the above-mentioned publications [47,49], carbonation was not observed, and may be due to the experimental conditions (number of equivalents of CDI, reaction time, temperature, adventitious presence of water). Nonetheless, the removal of the 2′,3′-carbonate protecting group can be easily performed under basic conditions [52,53].

**Scheme 5:**
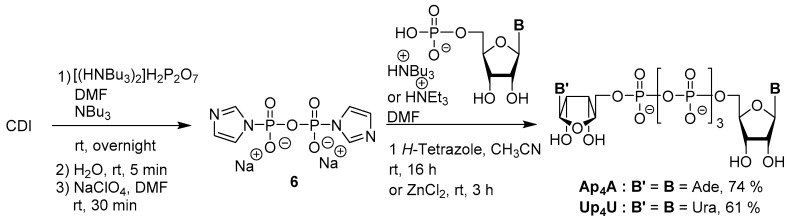
Np_4_Ns synthesis using *P^1^*,*P^2^*-di(1-imidazolyl)pyrophosphate **6 [54]**.

In 2011, a variant of the CDI method was developed by Yanachkov and co-workers [54], where the activation of pyrophosphate by CDI gave rise to *P^1^*,*P^2^*-di(1-imidazolyl)pyrophosphate (Scheme 5). Using ^13^C-labeled CDI and monitoring the reaction by ^31^P NMR (^13^C-^31^P and ^1^H-^31^P couplings), the authors proposed a reaction mechanism involving the fast formation of mixed anhydrides, which react slowly with imidazole to give diimidazolyl pyrophosphate. This activated pyrophosphate was isolated as its disodium salt **6** by precipitation with sodium perchlorate in acetone, and then reacted with AMP or UMP to afford the symmetrical 5′,5′-dinucleoside tetraphosphates Ap_4_A and Up_4_U in good yields (Scheme 5). Noteworthy, the use of 1*H*-tetrazole or zinc chloride during the second step allowed the reaction time to be shortened from 24–48 h to 3–16 h.

Interestingly, this approach allows the preparation of dinucleotide analogues, starting from modified pyrophosphate (the central oxygen being replaced with monofluoro, monochloro or dichloromethylene). These DNPs analogues were obtained in 70–80% yields.

While these methods involving phosphoromidazolides are straightforward and rapid, they all require dry conditions to prevent side-product formation and most often, use trialkylammonium salts. To avoid this drawback, our research group has recently developed a one-pot synthesis of dinucleotides, either in aqueous solution or mechanochemically, through the use of phosphorimidazolides intermediates [55,56]. These strategies will be detailed in Section 5 and are related to green chemistry approaches.

### 3.3. Synthesis via a Cyclic Trimetaphosphate Intermediate

**Scheme 6:**
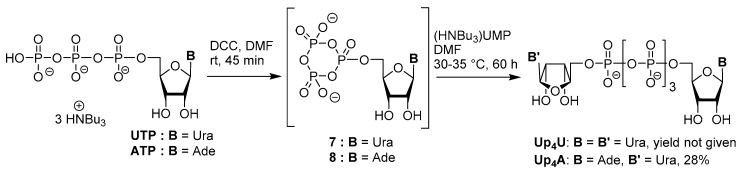
Synthesis of Np_4_Ns starting from nucleoside 5′-triphosphates (NTPs) [47].

The initial synthesis of trimetaphosphate esters from ATP was described by Khorana [57]. Later, Ng and Orgel reported that adenosine 5′-polyphosphates can react with 1-ethyl-3-(3-dimethylaminopropyl)carbodidimide (EDC) in water to form diadenosine 5′,5′-polyphosphates as the main products [58]. Yields were improved by performing the reaction in organic solvents such as DMF or DMSO, in the presence of DCC and using the tris or tetrakis(tri-*n*-butylammonium) salts of nucleotides [31,47]. The activation of nucleoside 5′-triphosphates (NTPs) with DCC proceeds via a cyclic nucleoside 5′-trimetaphosphate intermediates (**7–8**), which can further react with another NMP to produce the corresponding dinucleoside tetraphosphates (Scheme 6). Accordingly, Up_4_U and Up_4_A were obtained in low yields starting from UTP and ATP, respectively. One should note that *N,N’*-dicyclohexylurea (DCU) may be removed as a precipitate either after the first or the second step of the reaction. 

Similarly, the activation of UTP or ATP with DCC in anhydrous DMF, followed by the addition of the tris(tri-*n*-butylammonium) salt of UDP or ADP in the second step of the reaction, afforded Up_5_U and Ap_5_A, in 10–60% yields [8,49,50]. Reactions must be performed under strictly anhydrous conditions and long reaction times are required.

**Scheme 7:**
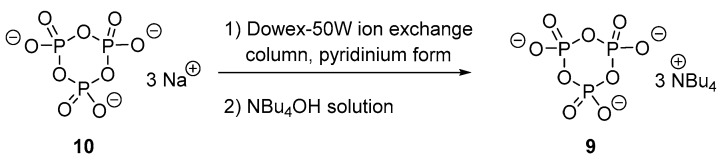
Preparation of the cyclic trimetaphosphate **9** [59].

In 2013, the Taylor research group reported a high-yielding synthesis of dinucleoside pentaphosphates (Np_5_N) using the tri(tetra-*n*-butylammonium) salt of cyclic trimetaphosphate **9** as a phosphorylating agent [59]. The latter was prepared in almost quantitative yield by conversion of the trisodium salt of trimetaphosphate **10** into its pyridinium form, and then by titration of the solution to pH 7.0 with a dilute solution of tetrabutylammonium hydroxide and freeze-drying (Scheme 7). 

**Scheme 8:**
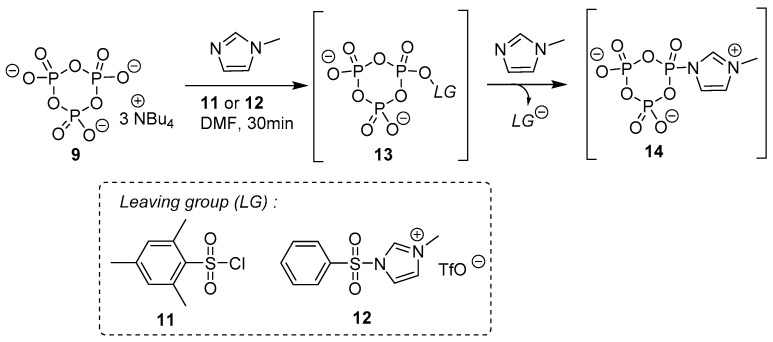
Activation of the cyclic trimetaphosphate **9** [59].

Then, the electrophilic reagent **9** was functionalized by a sulfonyl leaving group (LG) using *N*-methylimidazole (NMI) in the presence of either 2-mesitylenesulfonyl chloride **11** or 1-benzenesulfonyl-3-methyl-imidazolium triflate **12** in DMF (Scheme 8). Compound **11** was commercially available, whereas **12** was prepared in two steps starting from benzenesulfonyl chloride and imidazole [60]. Based on ^31^P NMR monitoring of the reaction, the authors proposed that the activation proceeds via a mixed anhydride **13**, which reacts with NMI to give rise to intermediate **14**.

**Scheme 9:**
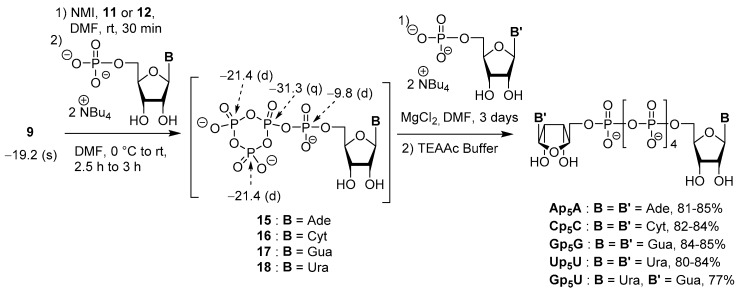
Synthesis of dinucleoside pentaphosphates via an activated cyclic trimetaphosphate [59]. ^31^P chemical shifts are indicated in ppm, together with their multiplicity.

Addition of sub-stœchiometric amounts of NMPs to the activated cyclic trimetaphosphate **9** allowed their conversion to intermediates **15–18**, as suggested by ^31^P NMR (Scheme 9). Addition of slight excess of the second NMP and anhydrous MgCl_2_ gave rise to the corresponding dinucleoside pentaphosphates. In the absence of Mg^2+^ ions, the reaction was extremely slow.

This method constitutes an interesting approach as it allows formation of symmetric as well as mixed Np_5_Ns in high yields, and uses inexpensive reagents (sodium cyclic trimetaphosphate, mesitylene chloride, NMI). It has recently been applied for the synthesis of dicaptides, used as substrates for polymerases [61]. However, it requires the preparation of the tetra-*n*-butylammonium salts of the cyclic trimetaphosphate and those of the nucleotides, reaction times are long (> 3 days), and must be performed under strictly anhydrous conditions.

### 3.4. Synthesis via a Phosphoromethylimidazolium Intermediate

Reacting the free acids of canonical nucleoside 5′-monophosphates with a large excess of NMI in the presence of triphenylphosphine/2,2′-dipyridyl sulfide as coupling agents and triethylamine in DMF or DMSO allowed dimerization to Np_2_N in about 85% yields [62]. Unsymmetrical dinucleotides could also be obtained, albeit in much lower yields, by using a equimolar amount of the two NMPs to be coupled. 

**Scheme 10:**
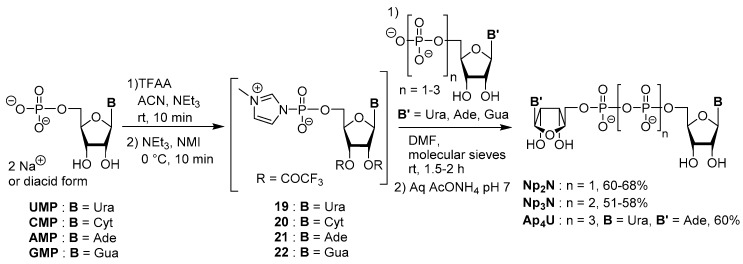
Synthesis of dinucleotides via phosphoromethylimidazolium intermediates [64].

Another methodology to provide symmetrical and mixed dinucleoside polyphosphates Np_n_N (*n* = 2–4) was inspired by the Bogachev procedure for the synthesis of deoxynucleoside 5′-triphosphates (dNTPs) [63]. It involves the reaction of nucleoside 5′-monophosphates-N-methylimidazolium as donors with an NMP, NDP or NTP to obtain dinucleoside di, tri and tetraphosphates (Scheme 10) [64]. These donors were prepared by treatment of the NMPs (disodium salts dihydrate or free acid hydrate) with a 16-fold excess of trifluoroacetic anhydride (TFAA) in acetonitrile (ACN) in the presence of excess triethylamine. This step resulted in the temporary protection of the hydroxyl and the amino (if present) groups and formation of a mixed anhydride, as shown by ^31^P NMR (δ = 2 ppm for the mixed anhydride formed within a few minutes). It offered the additional benefit of drying the starting materials without deleterious effects on the reaction outcome. However, in the case of GMP, these conditions resulted in the complete decomposition of the starting material. This limitation could be circumvented by using GMP in its tri-*n*-butylammonium form, together with reducing the number of equivalent of TFAA.

After removal of unreacted TFAA, the mixed anhydride was reacted with excess NMI in the presence of triethylamine to quantatively afford the highly reactive N-methylimidazolium salt donors **19–22** within 10 min. Formation of the N-methylimidazolium salts could also be easily followed by ^31^P NMR spectroscopy as these species exhibit chemical shifts of approximately −9 to −10 ppm. The crude acetonitrile solutions were used directly in the next step. Thus, addition of the donors solutions to the bis(tri-*n*-butylammonium) salts of the nucleotide acceptors in DMF (in acetonitrile precipitation of the acceptors was observed) led to partially protected products. The latter were treated with aqueous ammonium acetate to remove the protecting groups, providing the desired compounds. For dinucleotides involving guanine nucleotides, N,N-dimethylaniline was found to be beneficial. Using this procedure, up to nine dinucleotides were obtained in yields of 51–68%. It should be noted that this method cannot be used to activate nucleoside 5′-di- or triphosphates. While the reactions were performed under argon, the authors pointed out that vigorous drying of the donor precursors was not always necessary when preparing the dinucleoside di- and triphosphates. Advantages of this one-pot method are short reaction time and good yields. 

**Scheme 11:**
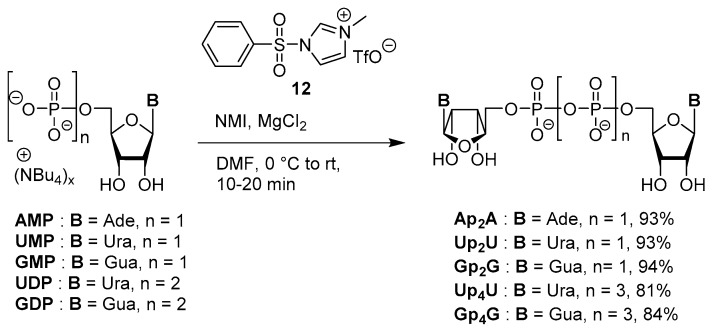
Synthesis of symmetrical Np_2_N and Np_4_N [60].

The same group also reported the synthesis of symmetrical di- and tetraphosphates by dimerization of nucleotides, using sulfonyl imidazolium salt **12** as a reagent (Scheme 11) [60,65]. The tetra-*n*-butylammonium salts of nucleoside 5′-mono or 5′-diphosphates were activated in DMF with 0.6–0.75 equiv of **12** in presence of 1–3 equiv of NMI and MgCl_2_ (Scheme 11). Dimerization of the nucleotides led to Np_2_N and Np_4_N in high yields.

**Figure 4:**
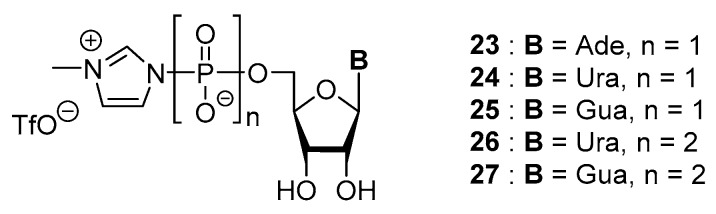
Phosphoromethylimidazolium intermediates [60].

^31^P NMR analyses showed that the reaction proceeds via the formation of the N-methylimidazolium salt intermediates **23–27** (Figure 4, ^31^P δ = −9.2 ppm), which are able to react with the remaining nucleotide to form the corresponding dimer. 

**Scheme 12:**
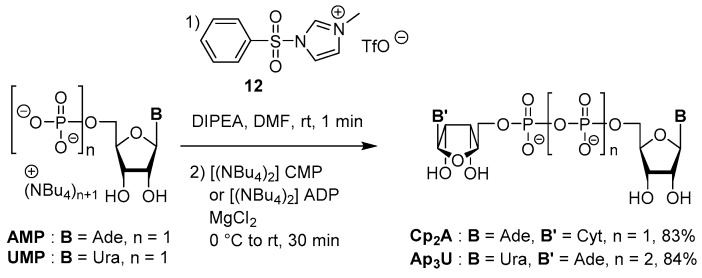
Synthesis of mixed dinucleotides [60].

Mixed dinucleotides could also be obtained in high yields, by introducing minor modifications into the protocol. Accordingly, activation was performed in the presence of a small excess of sulfonyl imidazolium salt **12**, and NMI was replaced by diisopropylethylamine (DIPEA) to prevent dimerization (Scheme 12). Moreover, the time of reaction of the first step was reduced to 1 min. Cp_2_A and Ap_3_U were obtained in similar yields starting from AMP and UMP, respectively. 

**Scheme 13:**
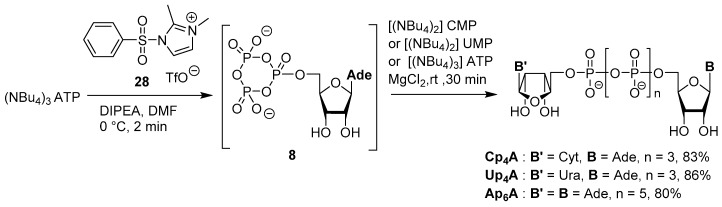
Synthesis of mixed dinucleotides starting from ATP [60].

Finally, mixed adenine dinucleotides were obtained in high yields by activating the tetra-*n*-butylammonium salt of ATP with sulfonyl imidazolium salt **28**, followed by reaction with a second nucleotide (Scheme 13). Investigation of the first step by ^31^P NMR revealed that the reaction proceeds via a cyclic adenosine trimetaphosphate intermediate **8**.

The advantages of this method using sulfonylimidazolium salts as key reagents, include short reaction times, access to a wide range of N_p_Ns (*n* = 2–6) and high yields. However, careful attention must be paid to maintain anhydrous conditions. 

### 3.5. Synthesis via a Phosphoropiperidate Intermediate

**Scheme 14:**
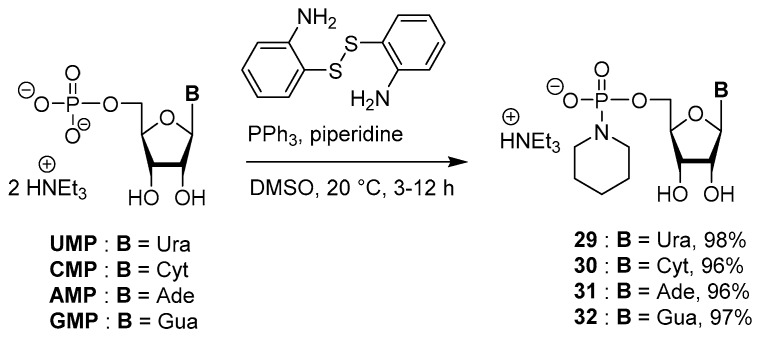
Synthesis of nucleoside 5′-phosphoropiperidates [66].

In 2014, the Sun research group developed an approach based on the activation of nucleoside 5′-phosphoropiperidates with 4,5-dicyanoimidazole (DCI) as activator. The phosphoropiperidates **29–32** were obtained beforehand from unprotected NMPs via a redox condensation involving triphenylphosphine and 2,2′-dithiodianiline (Scheme 14) [66].

**Scheme 15:**
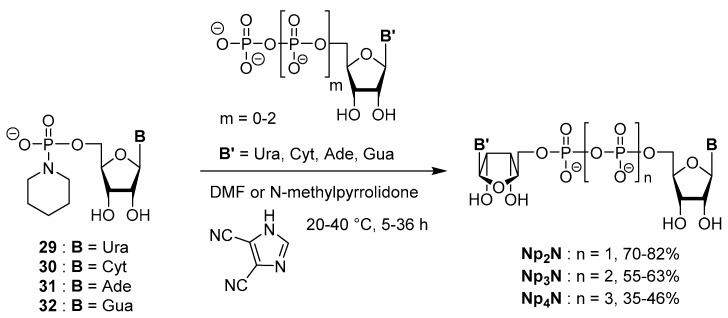
Synthesis of dinucleotides from nucleoside 5′-phosphoropiperidates [67,68].

As shown in Scheme 15, the reaction of nucleoside 5′-phosphoropiperidates with the tri-*n*-butylammonium salts of nucleotides (NMPs, NDPs, NTPs) in the presence of 2.5–3 equiv of DCI, led to the formation of more than 20 symmetrical or mixed dinucleoside di-, tri- and tetraphosphates [67,68]. Yields of Np_n_N’s dropped significantly along with the increase of the polyphosphate chain. 

Among the polar aprotic solvents, *N*-methylpyrrolidone exhibited the best solvency for the tri-*n*-butylammonium salts of both NDPs and NTPs, while DMF was preferred for NMPs. Considering the cost of nucleoside 5′-polyphosphates and their similar chromatography properties with the products, only 0.5 equiv of NDPs or NTPs were used. This P(V) method is an efficient one, allowing access to a large range of Np_n_Ns (*n* = 2–4) in short reaction times and good to high isolated yields.

**Scheme 16:**
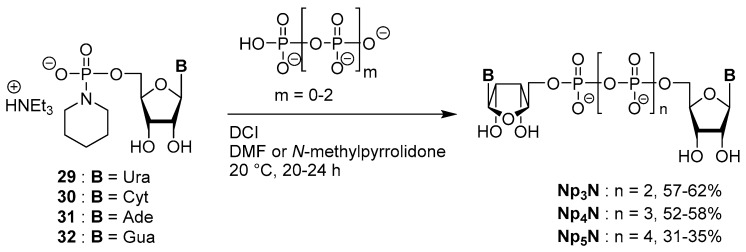
Synthesis of symmetrical NpN by dimerization [67,68].

Symmetrical Np_n_N (*n* = 3–5) could also be obtained by reacting nucleoside 5′-phosphoropiperidates with sub-stœchiometric amounts (0.35–0.4 equiv) of inorganic phosphorylating agents in the presence of excess DCI [69]. In this alternative protocol, bis(tetra-*n*-butylammonium) hydrogen phosphate, tris(tetra-*n*-butylammonium) hydrogen pyrophosphate or tris(tetra-*n*-butyl-ammonium) dihydrogen triphosphate were used instead of a nucleotide (Scheme 16). ^31^P NMR tracking experiments showed that pyrophosphate reacts first with the starting phosphoropiperidate to form an NTP, and then, with the remaining phosphoropiperidate to give rise to Np_4_N. This methodology was also adapted to the synthesis of nucleotides with a higher number of phosphate groups (such as Np_5_N), using inorganic triphosphate instead of pyrophosphate. 

Interestingly, this strategy can be adapted to the synthesis of nucleotide analogues such as dinucleoside *P^2^*,*P^3^*-(dihalo)methylene tetraphosphates, by replacing pyrophosphate with bisphosphonate reagents. 

## 4. Synthesis Based on P(III) Chemistry

Several methods involve P(III) reagents to provide phosphite or phosphoramidite intermediates, which are further oxidized to P(V). Initially developped for the synthesis of oligonucleotides, they have been adapted to dinucleotides by introducing minor modifications in the last steps of the reaction sequences. Because of the high reactivity of P(III) species, most of these approaches require the use of N-, and O-protecting groups on the starting nucleos(t)ides and strictly anhydrous conditions. 

### 4.1. Synthesis via a Salicylphosphite Intermediate

**Scheme 17:**
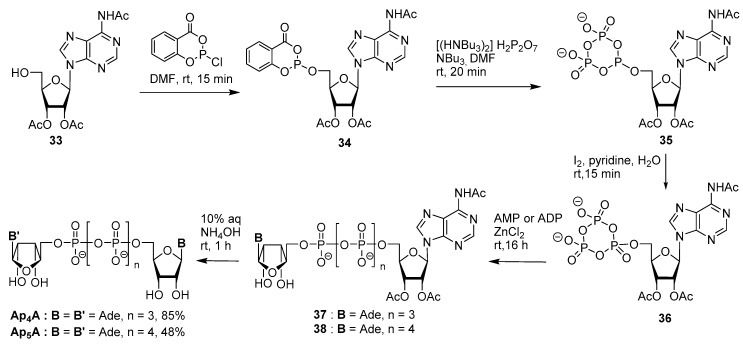
Synthesis of the symmetrical dinucleotides Ap_4_A and Ap_5_A by phosphitylation [71].

This strategy is based on the well-known Ludwig–Eckstein procedure for NTP synthesis [70], and allows the one-pot preparation of symmetric dinucleoside tetra- and pentaphosphates starting from protected nucleosides [71]. Phosphitylation of triacetyl adenosine **33** with 2-chloro-4*H*-l,3,2-benzo-dioxaphosphorin-4-one (salicylchlorophosphite) led to intermediate **34**, then addition of bis(tri-*n*-butylammonium) pyrophosphate gave rise to the cyclic intermediate P(III)–P(V) **35** (Scheme 17). Oxidation to the cyclic trimetaphosphate, intermediate **36**, followed by reaction with adenosine 5′-monophosphate (AMP) or adenosine 5′-diphosphate (ADP) in dry DMF in the presence of ZnCl_2_ as catalyst, afforded **37** and **38**, respectively. Removal of the acetyl groups under basic conditions, ion exchange to remove the Zn^2+^ cations and purification led to Ap_4_A and Ap_5_A as ammonium salts. It should be noted that symmetrical Ap_4_A was obtained in much better yield than Ap_5_A. 

**Scheme 18:**
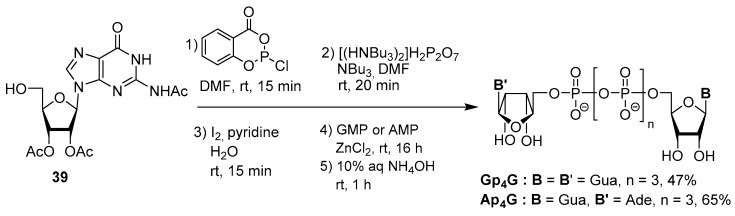
Synthesis of dinucleotides Gp_4_G and Ap_4_G by phosphitylation [71].

The guanosine derivatives Gp_4_G and Ap_4_G were prepared in a similar manner, starting from 2′,3′-*O*-2-*N*-triacetylguanosine **39** (Scheme 18).

### 4.2. Synthesis via a Cyclosaligenyl Phosphite Intermediate

**Scheme 19:**
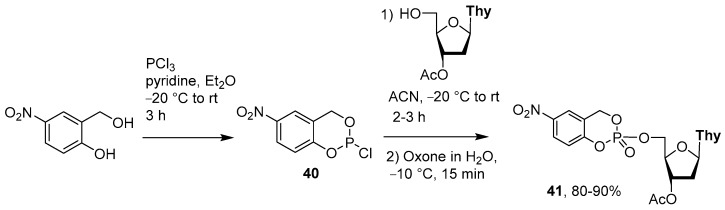
Synthesis of a *cyclo*Sal phosphotriester derivative of thymidine [74].

This strategy initially developed by Meier and co-workers for the synthesis of prodrugs of nucleotides, called *cyclo*Sal (for *cyclo*saligenyl) pronucleotides [72,73], has been applied to the synthesis of nucleoside 5′-polyphosphates, sugar nucleotides and mixed dinucleotides [74,75]. Phosphochloridite **40** was prepared from the corresponding salicyl alcohol and phosphorus trichloride (Scheme 19). Reaction with 3′-*O*-acetylthymidine followed by oxidation with oxone (2 KHSO_5_.KHSO_4_.K_2_SO_4_) afforded 5-nitro-*cyclo*Sal-3′-*O*-acetylthymidine phosphotriester **41**. 

**Scheme 20:**
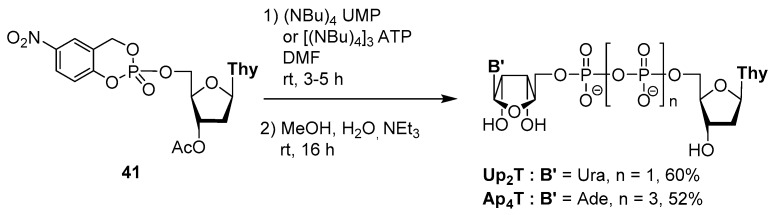
Synthesis of the dinucleotides Up_2_T and Ap_4_T [74].

This phosphotriester was too sensitive to moisture to be purified by chromatography. Therefore, the crude material (obtained after liquid-liquid extraction) was directly engaged in the next step, i.e., reaction with rigorously dried tetra-*n*-butylammonium UMP and tris(tetra-*n*-butylammonium) ATP in DMF (Scheme 20). After removal of the acetyl group and ion-exchange (*n*-NBu_4_^+^ to NH_4_^+^), Up_2_T and Ap_4_T were obtained in their ammonium form.

**Figure 5:**
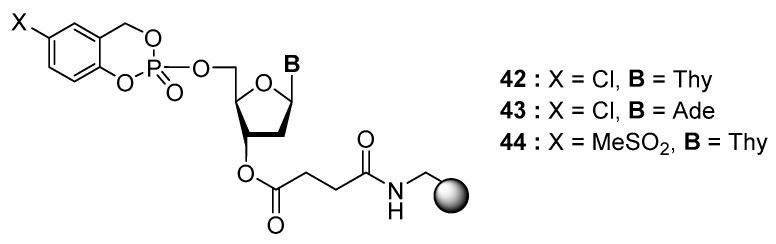
Structure of the supported nucleoside phosphotriesters [76].

This approach has been adapted to solid phase [76]. The key intermediates of the synthesis are the 5′-substituted *cyclo*Sal nucleotides linked to a polystyrene support through the 3′-OH group and a succinyl linker (**42**–**44**, Figure 5). 

**Scheme 21:**
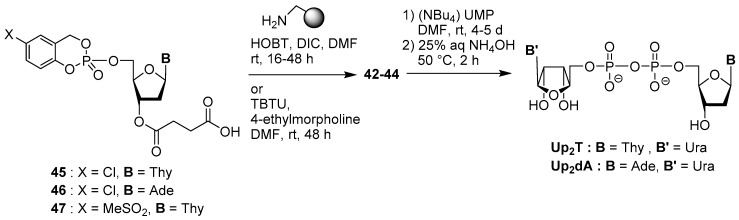
Solid-phase synthesis of dinucleoside 5′,5′-diphosphates [76].

Preparation of these intermediates started with the anchoring of the succinyl linker to the 5′-*O*-(4,4′-dimethoxytrityl)-protected nucleosides, and removal of the protecting group with trifluoroacetic acid (TFA). The 3′-*O*-succinylnucleosides were then treated with the appropriate chlorophosphites, followed by oxidation with oxone, to give rise to compounds **45**–**47** (Scheme 21). Coupling with the aminomethyl polystyrene support was achieved using 1-hydroxybenzotriazole (HOBt) and *N,N’*-diisopropylcarbodiimide (DIC) as coupling agents. Alternatively, 2-(1*H*-benzotriazole-1-yl)-1,1,3,3-tetramethyluronium tetrafluoroborate (TBTU) and 4-ethylmorpholine may be used. Conversion to Up_2_T and Up_2_dA was carried out by adding a large excess of tetra-*n*-butylammonium UMP, and then cleavage under basic conditions (70–78% purity, no overall yield was given). Compared to the solution phase synthesis, this supported approach is less straightforward and involves multiple steps.

### 4.3. Synthesis via a Supported Phosphite Intermediate

**Figure 6:**
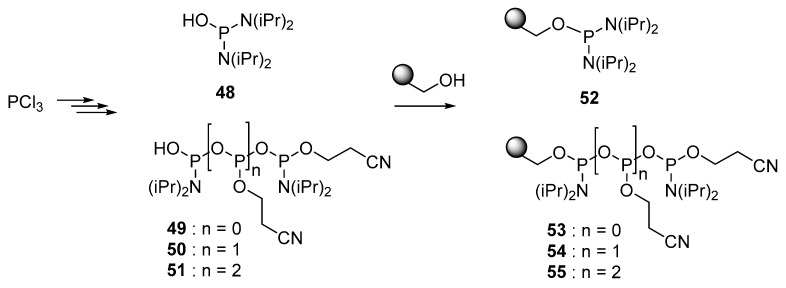
Synthesis of the immobilized phosphitylating reagents [77].

Another P(III) strategy combined with a solid-phase approach was reported by Ahmadibeni and Parang [77]. Using these polymer-bound phosphitylating reagents, symmetrical 5‘,5‘-dinucleoside mono, di, tri, and tetraphosphates were prepared. Compounds **48**–**51** were obtained in 2 to 5 steps starting from phosphorus trichloride (Figure 6). Coupling with aminomethyl polystyrene resin-bound *p*-acetoxybenzyl alcohol yielded four classes of supported-phosphitylating reagents **52**–**55** (Figure 6). 

**Scheme 22:**
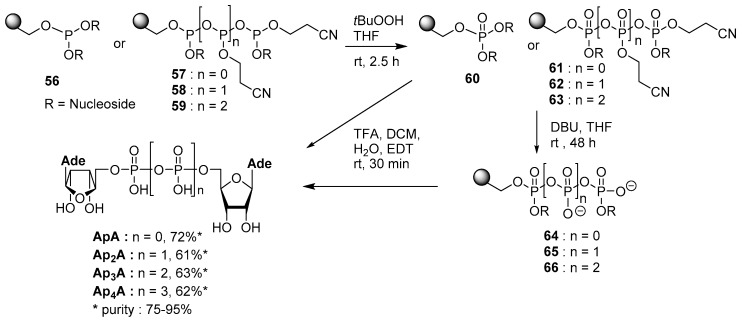
Synthesis of symmetrical dinucleotides using polymer-bound phosphitylating reagents. Yields are given for the adenine derivatives [77].

These supported reagents were reacted with unprotected nucleosides (e.g., thymidine, adenosine, cytidine, inosine or the analogue 3′-azido-3′-deoxythymidine) in the presence of 5-(ethylthio)-1*H*-tetrazole to afford compounds **56**–**59** (Scheme 22). Polymer-bound nucleotides underwent oxidation with *tert*-butyl hydroperoxide to **60**–**63**, then removal of the cyanoethoxy groups with 1,8-diazabicyclo[5.4.0]undec-7-en (DBU) was performed. Acidic treatment of **60** and **64**–**66** gave rise to the corresponding symmetrical dinucleotides in 59–78% yields starting from the supported reagents **52**–**55**.

This strategy allowed the synthesis of symmetrical dinucleotides starting from unprotected nucleosides without the need to purify the intermediates. It also offers the advantage of facile recovery of final products by filtration (trapped linkers on the resins). However, it requires the multistep synthesis of the supported polyphosphite reagents. It should be highlighted that part of this work could not be reproduced by others [78].

### 4.4. Synthesis via a 5′-H-Phosphonate Intermediate

**Scheme 23:**
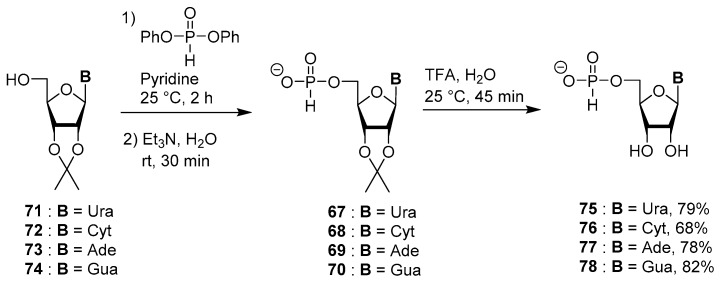
Synthesis of nucleoside 5′-*H*-phosphonates from the 2′,3′-protected nucleosides [79].

This one-pot strategy developed by Sun et al. [79], and based on their previous work on the synthesis of nucleoside triphosphates [80], involves the formation of a pyridinium phosphoramidate intermediate starting from a nucleoside 5′-*H*-phosphonate monoester. Nucleoside 5′-*H*-phosphonate monoesters **67**–**70** were obtained from 2′,3′-*O*-isopropylidene ribonucleosides **71**–**74** using diphenylphosphite as a reagent (Scheme 23). Then, removal of the isopropylidene group with aqueous TFA afforded 5′-*H*-phosphonate monoesters **75**–**78** in yields ranging from 68% to 82% over two steps.

**Scheme 24:**
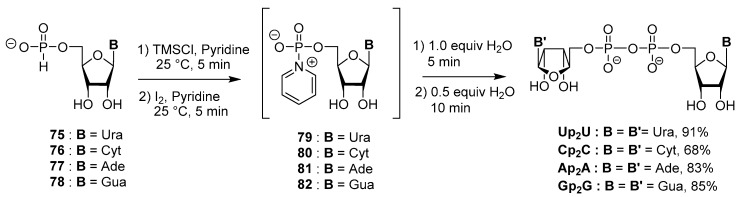
Synthesis of symmetrical dinucleotides from nucleoside 5′-*H*-phosphonates [79].

*H*-phosphonates were silylated with trimethylsilyl chloride (TMSCl) in pyridine, and in situ oxidized with iodine to generate the highly reactive zwitterionic pyridinium phosphoramidate intermediates **79**–**82** (Scheme 24). Indeed, even trace amounts of water may result in the formation of polyphosphate by-products. The authors hypothesized that if 50% of the phosphoramidate intermediate could be hydrolyzed with precise control, NMP generated in situ would react with the remaining phosphoramidate to afford Np_2_N as a major product. Accordingly, hydrolysis and coupling steps were optimized using ^31^P NMR tracking experiments and accomplished by two sequential additions of H_2_O in DMF. After purification and ion exchange, symmetrical dinucleoside diphosphates sodium salts were obtained in 68–91% yields starting from the nucleoside 5′-*H*-phosphonates. Advantages of this method are the short reaction time, simple purification procedure and high yields. However, it requires the preliminary synthesis of the *H*-phosphonate starting materials.

### 4.5. Synthesis via a Phosphoramidite Intermediate

**Scheme 25:**
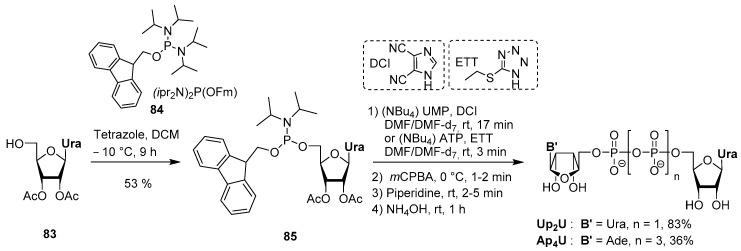
Synthesis of dinucleotides via a phosphoramidite intermediate [83].

In 2015, the Jessen research group developed a very efficient strategy based on an iterative phosphoramidite approach to access nucleoside 5′-polyphosphates [81,82] and some derivatives [83]. The phosphoanhydride bond is formed by coupling a phosphoramidite (the donor) to a phosphate (the acceptor). In contrast to the other P(III) methods, all steps were performed under ambient conditions, i.e., in open flasks and using non-dried solvents. Regarding of the synthesis of dinucleotides, 2′,3′-*O*-diacetyl uridine **83** was treated with phosphorodiamidite **84** in the presence of 1*H*-tetrazole to afford phosphoramidite **85** (Scheme 25). Addition of the tetra-*n*-butylammonium salt of UMP and DCI, followed by oxidation with *m*CPBA, treatment with piperidine, and cleavage of the acetyl groups under basic conditions, gave rise to Up_2_U as a mixed piperidinium/tetra-*n*-butylammonium salt. The mixed dinucleotide Ap_4_U was also obtained by using the tetra-*n*-butylammonium salt of ATP and 5-ethylthio-1*H*-tetrazole (ETT) as an activator in the second step of the reaction (Scheme 25). It should be noted that these experiments were performed on a small scale (10 mg).

**Figure 7:**
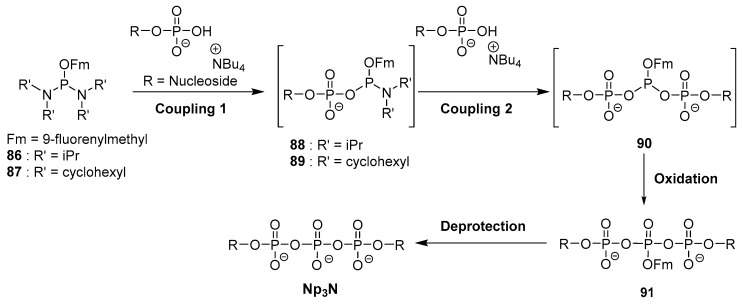
General approach for the preparation of symmetrical Np_3_Ns based on dimerization [84].

Symmetrical Np_n_Ns (*n* = 3, 5, 7) could also be accessed via chemoselective homologative dimerization of two phosphate monoesters with phosphorodiamidites [84]. The general strategy for the synthesis of dinucleoside triphosphates is shown in Figure 7. First, phosphordiamidites **86** and **87** (donors) were coupled with a NMP (the acceptor), affording terminal mixed phosphoric anhydride phosphoramidites **88**–**89** as unstable intermediates. Reaction with another phosphate monoester resulted in the formation of **90** containing a P^V^–P^III^–P^V^ bridge, which was then oxidized to yield **91**. The corresponding Np_3_N was obtained after a final deprotection step. 

**Scheme 26:**
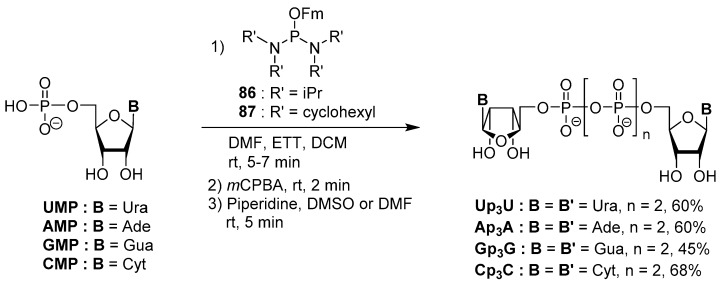
Synthesis of dinucleotides via dimerization of phosphates [84].

The tetra-*n*-butylammonium salts of the canonical nucleoside 5′-monophosphates were treated in DMF with 9-fluorenylmethyl-*N*,*N*,*N*’,*N*’-tetraalkyl phosphorodiamidites **86**–**87** in the presence of ETT, followed by oxidation with *m*CPBA (Scheme 26). Progress of the reaction was monitored by ^31^P NMR. The Fm-protected intermediates were isolated by precipitation with Et_2_O/hexane, treated with piperidine, and purified by anion exchange chromatography to isolate the symmetrical dinucleoside triphosphates as ammonium salts in good yields. 

This methodology was applied to the synthesis of symmetrical dinucleoside penta- and heptaphosphates by using as acceptor a NDP or a NTP, respectively. However, due to the water-sensivity of the intermediates, reactions had to be performed under anhydrous conditions, with a shorter reaction time and low temperature (coupling with ETT 30 s at 0 °C, oxidation 5 min at 0 °C). Accordingly, Ap_5_A and Ap_7_A were obtained in 50% and 18% yields, respectively. The chemoselective aspect of this method is a great advantage as it allows to prevent the use of nucleoside protecting groups. Compared to other procedures using P(III) chemistry, it offers attractive features, notably these rapid reactions can be run under ambient conditions.

## 5. Green Chemistry Approaches

Most of the reported methods present drawbacks, such as the use of non-volatile and toxic solvents (DMF, pyridine, *N*-methylpyrrolidone), high sensitivity to moisture, preparation of substrates or phosphorus reagents with lipophilic counterions due to solubility issues in organic media, anhydrous conditions (i.e., dry reagents and solvents), fastidious purification and possibly prior synthesis of the activated nucleotide substrates. An ideal procedure would meet the following requirements: Applicable to a wide range of substrates where protecting groups are not necessary, and giving rise to the products in short time and high yields.

**Scheme 27:**
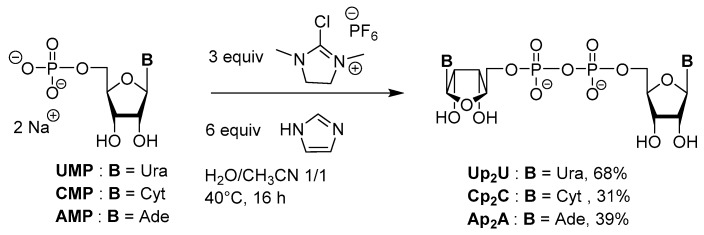
Preparation of symmetrical dinucleoside diphosphates in aqueous media [56].

In 2017, our research group reported a one-pot synthesis, in water medium, of nucleoside 5′-polyphosphates and dinucleotides starting from the corresponding NMPs [55,56]. This method uses 2-chloro-1,3-dimethylimidazolinium hexafluorophosphate (DMP) and imidazole as coupling reagents to activate the NMP into their phosphorimidazolides. Dinucleoside diphosphates were obtained by self-dimerization of the NMPs (Scheme 27).

**Scheme 28:**
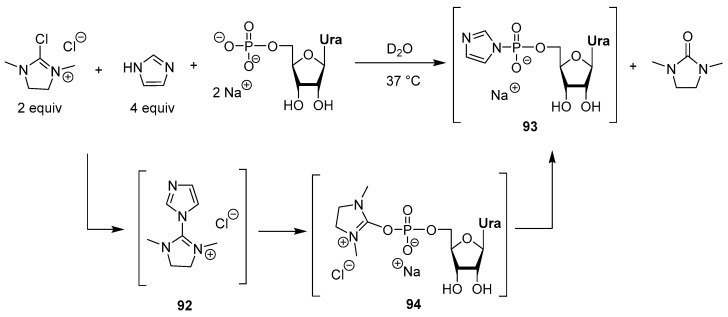
Proposed activation mechanism of UMP to UMP-Im by **90** [85].

Our work originated from the small-scale synthesis of NDP-sugars described by Tanaka et al. [85]. The authors performed the coupling of a sugar-1-phosphate with a nucleotide in D_2_O, using 2-chloro-1,3-dimethylimidazolinium chloride (DMC) and imidazole (Scheme 28). Based on ^1^H and ^31^P NMR spectroscopies, the reaction was shown to proceed via a 2-imidazolyl-1,3-dimethylimidazolinium chloride intermediate **92**, formed in situ by reaction of DMC and imidazole (Scheme 28). This key intermediate was able to activate an NMP into its corresponding phosphorimidazolide **93** (via intermediate **94**), which then reacted with a sugar-1-phosphate salt to form a sugar nucleotide. 

The main advantage of these reactions, which occur in D_2_O [85] or in H_2_O/CH_3_CN [55,56] instead of the usual organic solvents, is that the NMPs are used in their commercially available and water-soluble forms, i.e., sodium and potassium salts. This greatly simplifies the handling of the reaction. Moreover, protecting groups on the nucleotide are not required and no carbonation of ribonucleotides are observed, in contrast to the use of CDI.

**Scheme 29:**
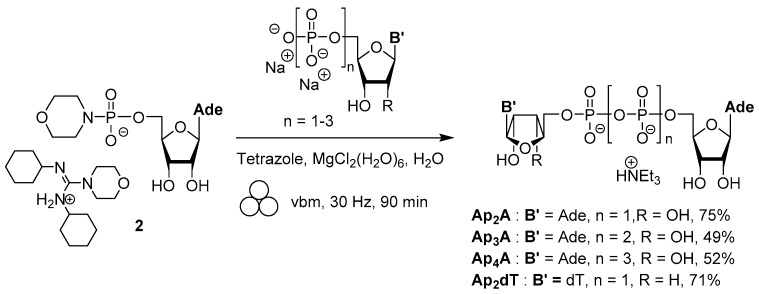
Ball-milling route using commercially available nucleotide salts and morpholidate reaction partners [92].

Alternatively, mechanochemistry has emerged in the field of nucleosides and, to a lesser extent, nucleotides [86]. This technique enables solid/solid reactions through mechanical grinding under solvent-free conditions [87,88,89,90,91]. In 2011, Ravalico et al. reported the mechanosynthesis of dinucleotides, namely Ap_n_A (with *n* = 2–4), Ap_2_dT and nicotinamide adenine dinucleotide (NAD^+^), starting from adenosine 5′-phosphoromorpholidate **2**, in the presence of magnesium chloride, 1*H*-tetrazole as the acidic promoter and water (Scheme 29) [92]. Although advantageous, this methodology requires the use of costly reagents, namely tetrazole and the phosphoromorpholidate substrate, the latter may be prepared beforehand in solution using conventional solution synthesis [45].

**Scheme 30:**
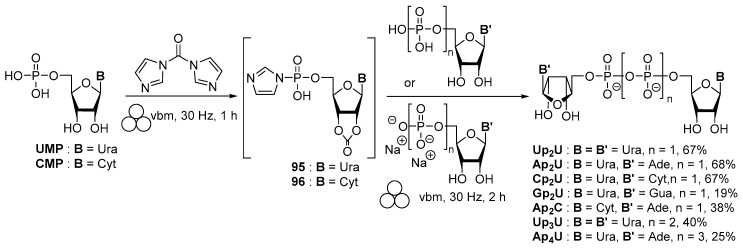
Mechanochemical approach to obtain DNPs starting from NMPs [93].

In the course of our investigations on new strategies for phosphoanhydride bond formation, we also investigated the ball-milling technique [93]. To begin with, activation of UMP was performed according to the conditions previously reported in water medium (DMP/imidazole) [55,56], without adding any solvent in a vibratory ball-mill (vbm) at 30 Hz. A complete conversion to Up_2_U was obtained within 30 min, suggesting that UMP was activated to its phosphorimidazolide and reacted promptly with the remaining UMP to form the dimer. Alternatively, we tested the use of CDI, which has recently gained interest in the field of mechanosynthesis due to the safety of its by-products (i.e., imidazole and carbon dioxide), its efficacy in *N*-acylation reactions and relatively low cost [94,95,96,97]. Activation of UMP (acidic form) to the corresponding 5′-phosphorimidazolide 2′,3-carbonate **95** was complete by grinding, for 1 h at 30 Hz, in the presence of 4 equiv. of CDI and acetonitrile (0.3 µL.mg^−1^) as a liquid assistant (Scheme 30). The absence of reactivity observed using the disodium salt of UMP suggests that the acid form is required to protonate the imidazole of CDI and to facilitate its substitution by the phosphate monoester. This activation step was applied successfully to several ribonucleotides and a 2′-deoxyribonucleotide (dTMP).

In the second step, a nucleoside mono, di or triphosphate was added onto the activated NMP **95**–**96** in order to form the desired DNPs (Scheme 30). Thus, the mixed dinucleoside 5′,5′-diphosphates were successfully obtained by adding a slight excess of a NMP and a small amount of acetonitrile (0.6–0.95µL/mg) in the jar, and then ball-milling for 2 h at 30 Hz. Remarkably, the second grinding step also removed the carbonate protection. The reaction was less efficient with GMP, UDP and ATP, and, thus, required a larger excess of reagent to form the corresponding dinucleotides Gp_2_U, Up_3_U and Ap_4_U, respectively. This user-friendly mechanochemical approach only requires a slight excess of reagents and limits the formation of side products. While isolated yields of symmetrical and mixed dinucleoside polyphosphates are similar to other approaches, the set-up of the experiments and the workup are greatly simplified, saving a considerable amount of time. In addition, compared to the previously reported mechanochemical synthesis [92], this one-pot two steps reaction starts from NMPs, which are readily available.

In addition to their simplicity, the mechanochemical approaches alleviate issues associated with the low solubility of reagents in solution phase synthesis. Therefore, nucleotides and inorganic salts can be used in their commercially available sodium or potassium forms.

## 6. Purification and Characterization

### 6.1. Purification

In this section, we provide a brief overview of the purification procedures for dinucleotides. Due to their high polarity, they cannot be separated by normal-phase chromatography like most organic compounds. In addition, the byproducts or side products formed during synthesis share similar characteristics, resulting in a challenging separation from the desired compounds. The two main purification methods, reported in Table 1, use ion-exchange or reverse-phase (RP) chromatography.

The first type of ion-exchange support is positively charged, such as diethylaminoethyl (DEAE) Sephadex, and allows the chemical species to elute according to their number of charges. Elution is often performed using a gradient of triethylammonium hydrogen carbonate (TEAB) or ammonium hydrogen carbonate (NH_4_HCO_3_) buffers. Regarding C_18_ reverse-phase chromatography, either preparative or semi-preparative high pressure liquid chromatography (HPLC) or chromatography on RP-18 silica gel may be used. Compounds are often eluted using a gradient from an aqueous buffered solution, such as triethylammonium acetate (TEAAc) or TEAB, to acetonitrile or methanol. Alternatively, gel filtration was described for the purification of symmetrical Np_2_N. Then, the samples are usually freeze-dried and converted to their sodium form by ion exchange using a Dowex-50W-Na^+^ resin. The fact that purification is a time-consuming step is one of the reasons that pushed some research groups to develop solid phase strategies [76,77]. Indeed, those methods were designed in order to simplify purification of the final products, which can be collected by simple filtration. However, the recovered materials showed moderate purity and an additional purification step was required [76]. Consequently, solution-phase approaches are still predominant.

### 6.2. Physico-Chemical Properties

NMR, UV spectroscopy, and mass spectrometry (MS) are the main tools for the analysis of dinucleotides. Since the sugar and the phosphate moieties have no significant absorption above 230 nm, dinucleotides exhibit UV absorption profiles similar to those of their parent nucleosides with λ max values close to 260 nm [1]. MS analysis of nucleotides and derivatives such as dinucleotides has been extensively reviewed by Banoub et al. [98]. Alternatively, ^31^P NMR is a very convenient tool to monitor reaction courses, characterize reaction intermediates, and study the conformation of dinucleotides and final compounds [1,50]. The chemical shift (δ) for most endogenous dinucleotides covers −8 to −24 ppm, and exhibits pH and counter ion dependence [1]. Table 2 summarizes the literature data for representative N_p_N (*n* =1–7). In ^1^H-decoupled ^31^P NMR, the phosphorus atoms closest to the nucleosides are characterized by a singlet at approximately −10 ppm, while the phosphorus atoms inside the polyphosphate chain exhibit resonance signals around −22 ppm. 

Interestingly, the study of dinucleotides using NMR techniques (^1^H, ^13^C and ^31^P) can determine the conformation (*syn*/*anti*, sugar puckering), nucleobase properties (tautomerism, H-bonds and π-stacking interactions), as well as the anomeric α/β configuration. Such data are crucial for understanding the structure–activity relationship of either natural or synthetic dinucleotides as enzyme inhibitors and receptor ligands, and the design of potent therapeutic agents based on a dinucleotide scaffold. In this regard, Stern et al. have performed conformational studies on physiologically active Np_n_N′ sodium salts (N = A, G, U, C; N′ = A, G, U, C; *n* = 2–5) in neutral aqueous solution, using NMR and computational techniques [50,98]. While no predominant conformation was observed for the ribose moiety in any of the studied dinucleotides, natural dinucleoside polyphosphates were more frequently found in a folded (stacked) rather than an extended conformation. Purine dinucleotides showed greater stacking interactions than pyrimidine dinucleotides. In addition, dinucleotides with longer phosphate chains were found to have weaker stacking interactions. Crystal structures of Ap_4_A sodium salt [99], and some dinucleotides bound to kinases or hydrolases are available [100,101]. In protein-bound dinucleotides, the polyphosphate chain adopts various conformations: extended [100,101,102,103], S-shaped [101] or folded [100]. In some cases, the protein-bound dinucleotides coordinate with a Mg^2+^ ion. 

Finally, Chen and Kohler investigated the dynamics of excited electronic states formed by UV excitation of the several diadenosine polyphosphates by femtosecond transient absorption (TA) spectroscopy [104]. They found that the excimer states seen in TA experiments on nucleobase dimers are only observed in π-stacked conformations, but the lifetimes of these states are insensitive to how the stacked bases are oriented.

## 7. Conclusions

Naturally occurring dinucleoside 5′,5′-polyphosphates are involved in a variety of cellular processes. Among them, symmetrical purine-containing dinucleotides are the most known representatives, whereas pyrimidine-containing and mixed derivatives are still intensively studied. Therefore, the development of synthetic methods required to obtain these compounds in few steps, good yields, high purity and comfortable amounts generated interest from the chemists and remains a field of intensive research. Herein, we have focused our attention on the synthesis of dinucleoside 5′,5′-polyphosphates, either symmetrical or mixed, and including non-modified pyrophosphate bonds. These methods may involve P(III) and/or P(V) intermediates and solution-phase or supported chemistry. P(V) synthetic approaches are usually based on the activation of a 5′-nucleotide using inexpensive reagents but require the conversion of the substrates, as well as the phosphate reactants, into the corresponding tri- or tetra-alkyl salts due to solubility issues. P(III) synthetic approaches are often effective processes. However, the presence of protecting groups on the nucleoside is often mandatory, thus increasing the overall number of steps. In most cases, strictly anhydrous conditions are essential and the length of the poly-phosphate chain affects the yields of the synthesis. Recently, environmental friendly alternatives were investigated and may constitute an area of substantial and growing interest for the preparation of these derivatives in the coming years.

## Figures and Tables

**Table 1:** Main methods to isolate dinucleotides.

Types of Chromatography	Eluants	References
**Anion-exchange**		
DEAE-Sephadex^®^	0.02 M TEAB, pH 8; 10% ACN	[54]
TOYOPEARL^®^ DEAE-650M	0.2 M TEAB, pH 8; 10% ACN	[54]
DEAE-Sephadex^®^	Gradient of aq. NH_4_HCO_3_	[67,69]
Q Sepharose^®^ Fast Flow	Gradient of aq. NH_4_HCO_3_	[83,84]
DEAE Sepharose^®^ Fast Flow	Gradient of aq. NH_4_HCO_3_	[83,84]
**C-18 reverse-phase**		
HPLC	0.01 M NH_4_HCO_2_, pH 4, 50% MeOH	[46]
Semi-preparative HPLC	TEAAc pH 7 or 9; 6–12% ACN	[59,60,64]
Preparative HPLC	0.1 M NH_4_HCO_3_; ACN	[71]
Preparative HPLC	TEAB pH 7.8; 65% ACN	[92]
RP-18 silica gel column	Water	[74]
RP-18 silica gel column	0.1 M TEAB, pH 7.5; 20% ACN	[55,56]
RP-18 silica gel column	0.1 M TEAAc, pH 7; 5–10% ACN	[93]
**Gel filtration**		
Sephadex^®^ LH-20	Deionized water	[79]

**Table 2:** ^31^P Chemical shift ranges of dinucleoside polyphosphates in D_2_O.

Np_n_N	δ Ranges	References
Np_2_N’	1 singlet (*P*^1^ + *P^2^*) from −9.6 to −11.5 ppm	[47,49,50,53,56,60,64,67,74,76,77,79,83,93]
Np_3_N	1 doublet (*P*^1^ + *P^3^*) from −9.2 to −11.7 ppm 1 triplet (*P^2^*) from −21.0 to −23.4 ppm	[47,49,50,54,60,64,67,69,84,93]
Np_4_N	1 multiplet or 1 broad singlet or 1 doublet (*P*^1^ + *P*^4^) from −8.8 to −11.5 ppm	[47,49,50,54,60,64,67,69,71,74,93]
	1 multiplet or 1 br singlet or 1 doublet (*P^2^* + *P^3^*) from −20.6 to −23.1 ppm	
Np_5_N	1 multiplet or 1 br singlet or 1 doublet (*P*^1^ + *P^5^*) from −8.7 to −11.4 ppm	[49,50,59,69,71,84]
	1 multiplet or 1 br singlet or 1 doublet (*P^2^* + *P^3^ + P^4^*) from −20.2 to −22.5 ppm	
Np_6_N	1 doublet at −10.1 ppm (*P*^1^ + *P^6^*) 1 multiplet at −21.7 ppm (*P ^2^*+ *P^3^ + P^4^ + P^5^*)	[49]
Np_7_N	1 broad singlet or 1 doublet from −10.1 to −11.6 ppm (*P*^1^ + *P^7^*)	[49,84]
	1 multiplet from −21.7 to −23.5 ppm (*P^2^* + *P^3^ + P^4^ + P^5^ + P^6^*)	
